# Supplementary material for: Wastewater metaproteomics: tracking microbial and human protein biomarkers
Source: ISME Commun. 2025 Dec 18;6(1):ycaf243. doi: 10.1093/ismeco/ycaf243 (PMC12815272; doi:10.1093/ismeco/ycaf243)
Supplement: Tugui_et_all_2025_WW_metaproteomics_SI_DOC_ycaf243 [file tugui_et_all_2025_ww_metaproteomics_si_doc_ycaf243.pdf]

Supplementary information material to:
Wastewater metaproteomics: tracking microbial and human protein biomarkers

Claudia G. Tugui, Filine Cordesius, Willem van Holthe, Mark C.M. van Loosdrecht and Martin Pabst*
Department of Biotechnology, Delft University of Technology, 2629 HZ Delft, The Netherlands.
*Contact: m.pabst@tudelft.nl

TABLE OF CONTENTS

SI Figure 1A: LDA analysis of potential pathogens Page 2
SI Figure 1B: LDA analysis of human proteins Page 2
SI Figure 2: Proteins in HP and UT related to different types of cancers Page 3
SI Figure 3A: PCA analysis of human proteins identified in HP Page 4
SI Figure 3B: PCA analysis of human proteins identified in UT Page 4
SI Figure 4A: PCA analysis of metaproteome (excluding human proteins) identified in HP Page 5
SI Figure 4B: PCA analysis of metaproteome (excluding human proteins) identified in UT Page 5
SI Table 1: Proteins associated with different types of cancers in HP and UT Page 6
SI Figure 5: N-glycosylation mass modification profile on human proteins Page 7
SI Figure 6: Boxplot of total protein abundances per location Page 7
SI Figure 7: Proteins with significantly different abundance between locations Page 8

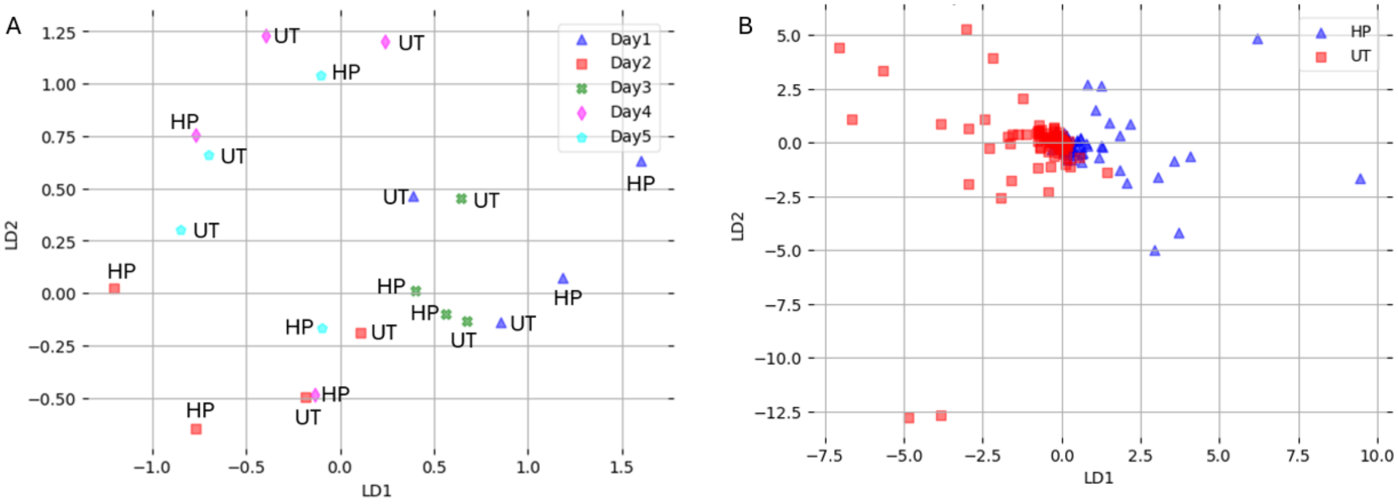

SI Figure 1. A) LDA analysis on potential pathogens (as defined by WHO report) discovered in wastewater samples based on sampling days. **B)** LDA analysis on the detected human proteome based on location (UT = Utrecht, HP = Harnaschpolder).

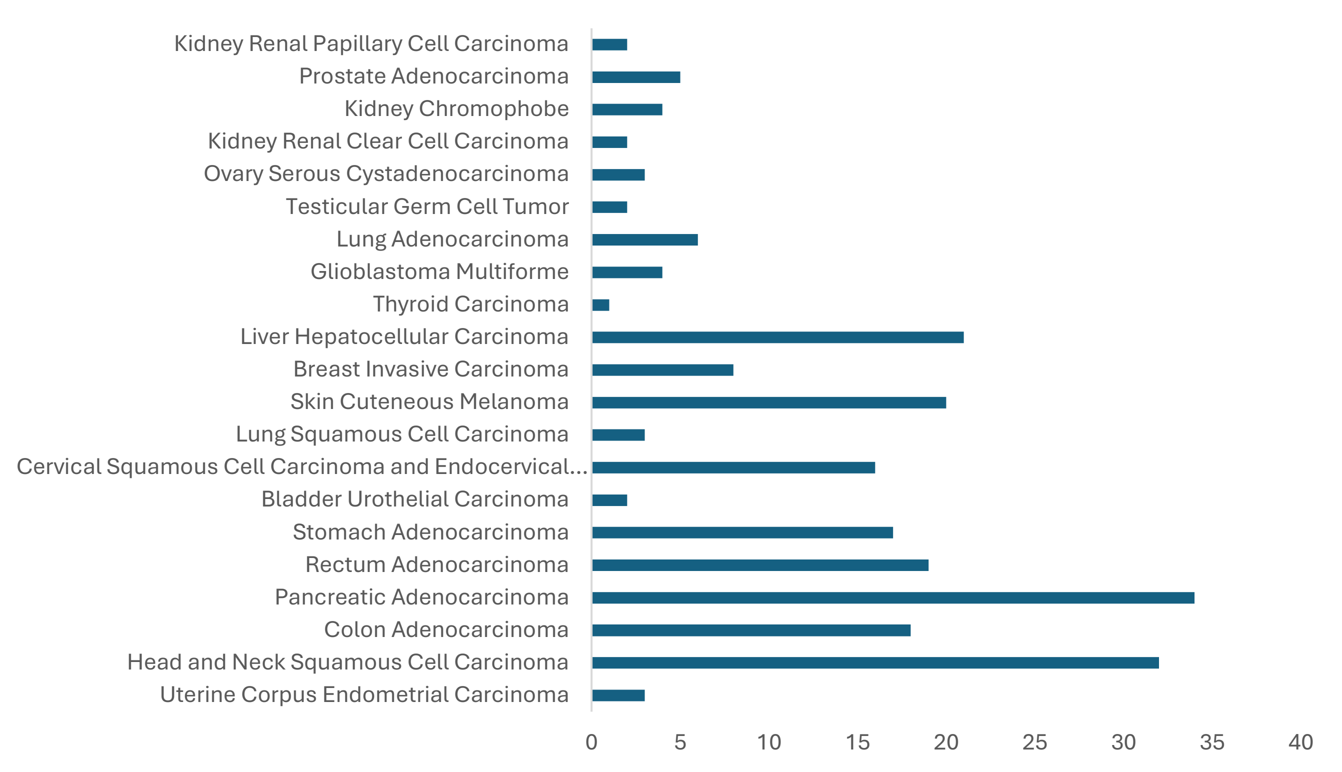

SI Figure 2. Number of human proteins with potential clinical relevance (e.g. biomarkers) for different types of cancer by both locations.

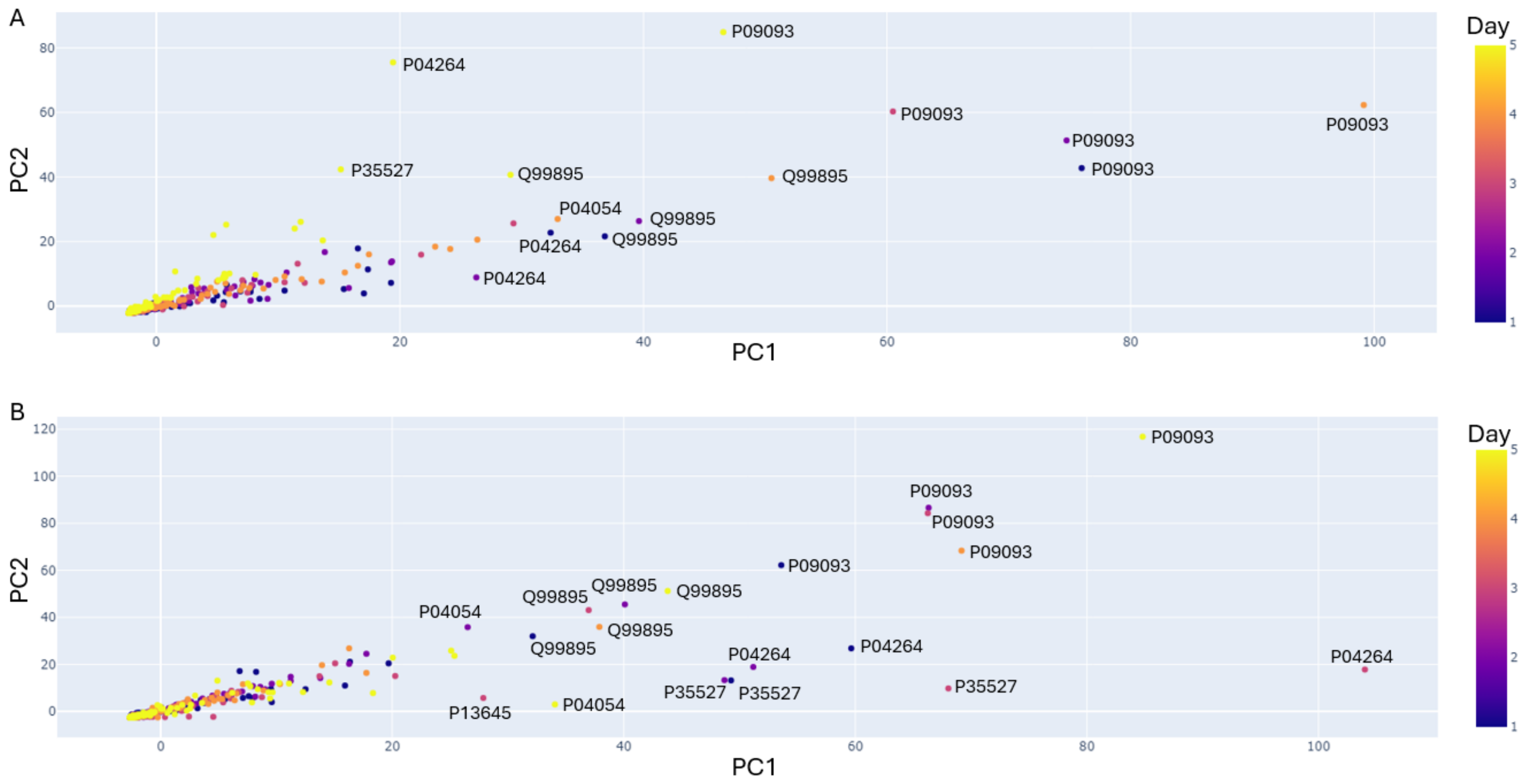

SI Figure 3. PCA analysis of the detected human proteome for HP (Graph A) and UT (Graph B). Outliers are annotated by their UniProtKB accession number. The color of the dot represents the day of sampling.

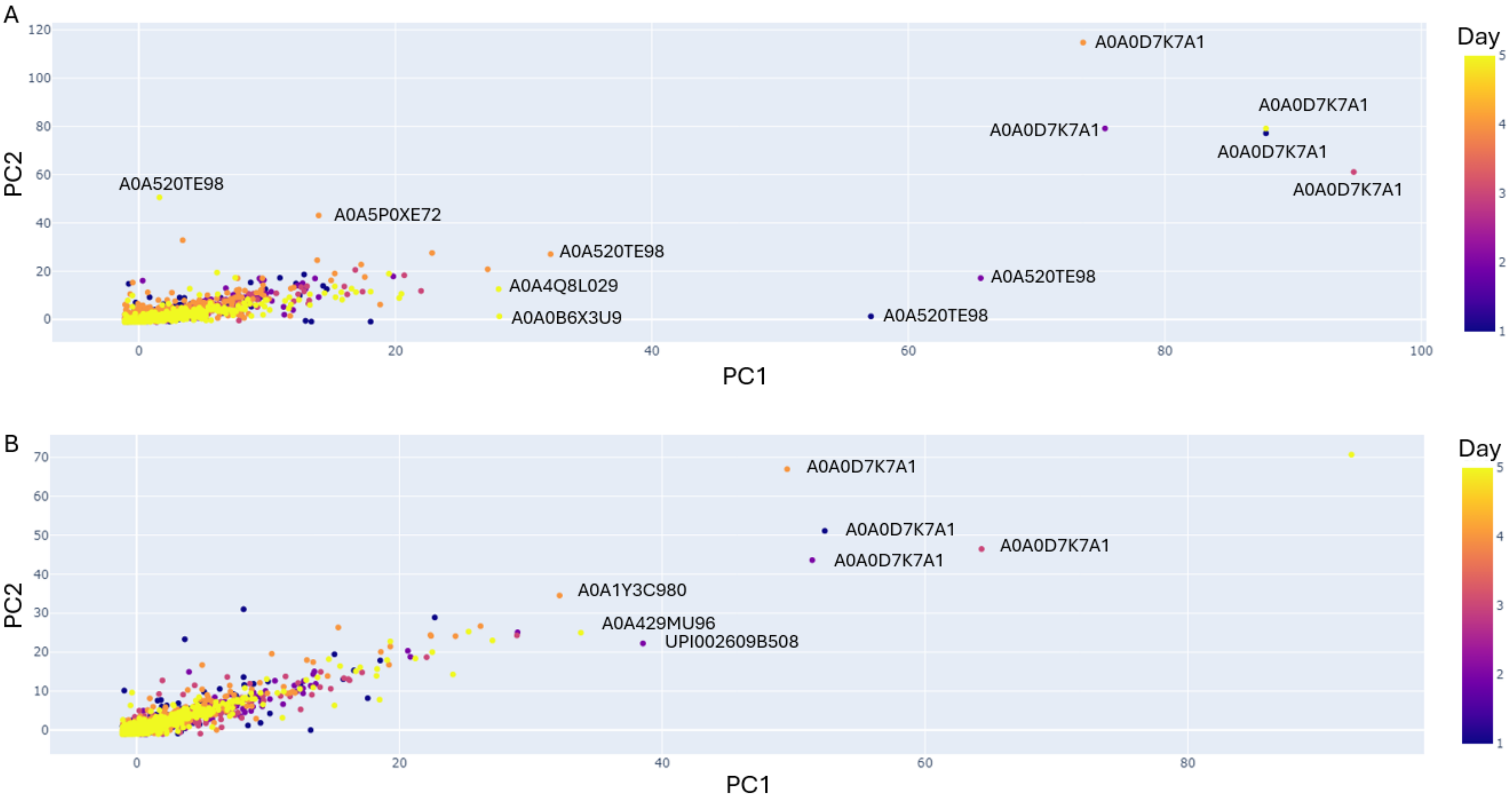

SI Figure 4. PCA analysis of the detected microbial metaproteome for HP (Graph A) and UT (Graph B). Outliers are annotated by their UniProtKB accession number. The color of the dot represents the day of sampling.

SI Table 1. List of proteins potentially related to different types of cancer, as classified by the Human Protein Atlas (UT = Utrecht, HP = Harnaschpolder).

Cancer type	UniProtKB accessions of potentially related proteins
Uterine Corpus Endometrial Carcinoma	Q14508, P01036, P20160
Head and Neck Squamous Cell Carcinoma	P04264, P15924, P13645, P08779, P02533, P04259, P13647, Q04695, Q02413, P13646, P29508, Q8N1N4, P06702, Q01546, Q5T749, Q08554, Q08188, Q9NZT1, P04083, Q01469, P31944, P31151, Q96P63, P05109, P35609, Q13835, P22532, A8K2U0, P01040, Q15517, P63316, P55000
Colon Adenocarcinoma	Q9Y6R7, Q02817, A8K7I4, Q9UGM3, Q16819, Q8WWA0, P35030, P56470, O60844, P06731, P40879, P05451, P00915, Q14002, P13688, Q12864, Q9H3R2, Q6UX06
Pancreatic Adenocarcinoma	Q02817, P04746, P19961, A8K7I4, Q9UGM3, Q86UP6, P08861, P09093, P07477, P55259, P16233, P07478, Q6GPI1, P08217, P15085, P98088, Q8WWA0, Q99895, P48052, P04054, P98073, Q16820, P35030, P56470, P05451, Q6W4X9, P04118, Q03403, P19835, P02766, Q9H3R2, P31025, Q9HD89, Q6UX06
Rectum Adenocarcinoma	Q02817, A8K7I4, Q9UGM3, Q16819, Q8WWA0, Q8WWU7, P35030, P56470, O60844, P06731, P40879, P00915, Q14002, P13688, Q12864, P22748, P40199, Q9H3R2, Q6UX06
Stomach Adenocarcinoma	Q02817, Q9UGM3, P14410, P98088, Q8WWA0, P05164, P98073, P35030, P61626, P56470, P05451, P80188, Q12864, Q6W4X9, Q03403, Q9H3R2, Q6UX06
Bladder Urothelial Carcinoma	P13646, P31944
Cervical Squamous Cell Carcinoma and Endocervical Adenocarcinoma	P13647, Q04695, Q14CN2, P13646, P29508, P48594, Q8N1N4, P06702, Q9NZT1, Q01469, P31944, Q96P63, P05109, Q13835, P01040, Q9HCY8
Lung Squamous Cell Carcinoma	P13646, Q96P63, Q13835
Skin Cuteneous Melanoma	P04264, P35527, P35908, P13645, P04259, Q86YZ3, Q02413, Q5D862, Q8N1N4, P05090, Q5T749, Q08554, Q9NZT1, P31944, P31151, Q96P63, O14556, Q15517, P20930, P55000
Breast Invasive Carcinoma	P02788, P25311, P12273, Q96DA0, P00709, P01036, P81605, P02814
Liver Hepatocellular Carcinoma	P02768, P02787, P01009, P09923, O43895, P25311, Q6UWV6, P01011, P02760, P01008, P00738, P56470, P05089, P02763, O95497, P05154, P00734, P02790, P02766, P02750, P05155
Thyroid Carcinoma	P61916
Glioblastoma Multiforme	P01011, P35609, P10153, P59665
Lung Adenocarcinoma	P0DTE8, Q9UGM3, P98088, Q8WWA0, P40199, Q9HD89
Testicular Germ Cell Tumor	Q8WWU7, Q6PEY2
Ovary Serous Cystadenocarcinoma	P0DTE8, Q14508, P31025
Kidney Renal Clear Cell Carcinoma	P09923, P07911
Kidney Chromophobe	P07911, P06870, P24855, P01133
Prostate Adenocarcinoma	P27487, P25311, P15309, Q96DA0, P14555
Kidney Renal Papillary Cell Carcinoma	P15144, Q9BYE9

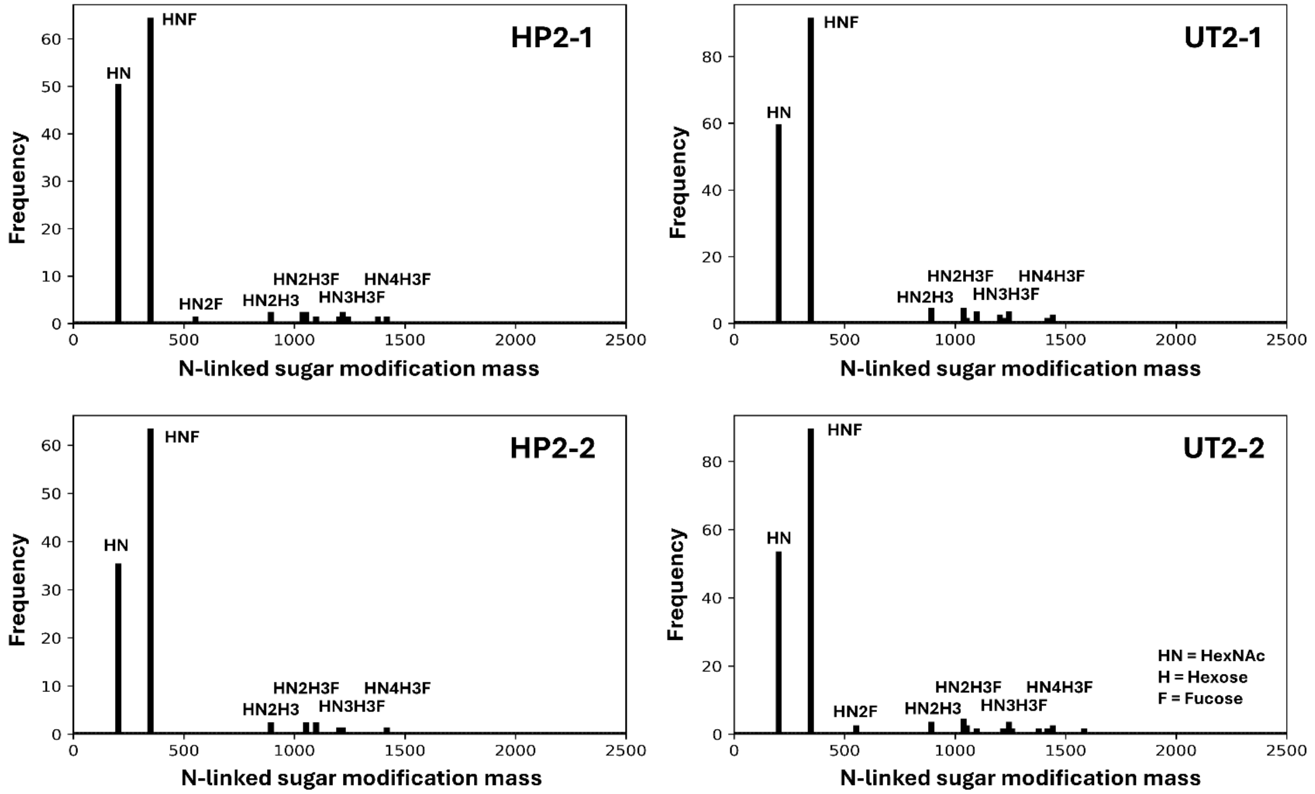

SI Figure 5. Frequency of observed N-glycan mass modifications on human proteins detected in wastewater samples from Harnaschpolder (HP2, collected 12 Dec 2022; suffix 1 = technical replicate 1, suffix 2 = technical replicate 2) and Utrecht (UT2, collected 5 Dec 2022; suffix 1 = technical replicate 1, suffix 2 = technical replicate 2). The modification profile shows predominantly trimmed glycans, mainly single HexNAc or HexNAc-Fuc residues, likely due to exo- and endoglycosidase activity present in wastewater.

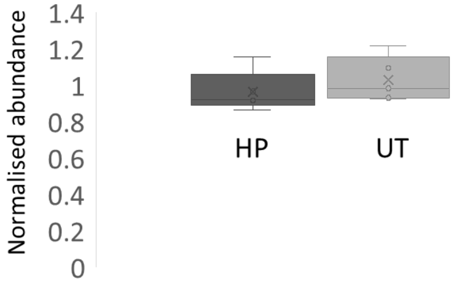

SI Figure 6. Boxplot of total protein abundances per location. The left box represents wastewater from Harnaschpolder (HP) and the right box from Utrecht (UT). Boxes show the interquartile range (middle 50% of values), whiskers indicate the minimum and maximum values, the horizontal line marks the median, and the cross indicates the mean. All abundance values were normalized to the overall average across all samples.

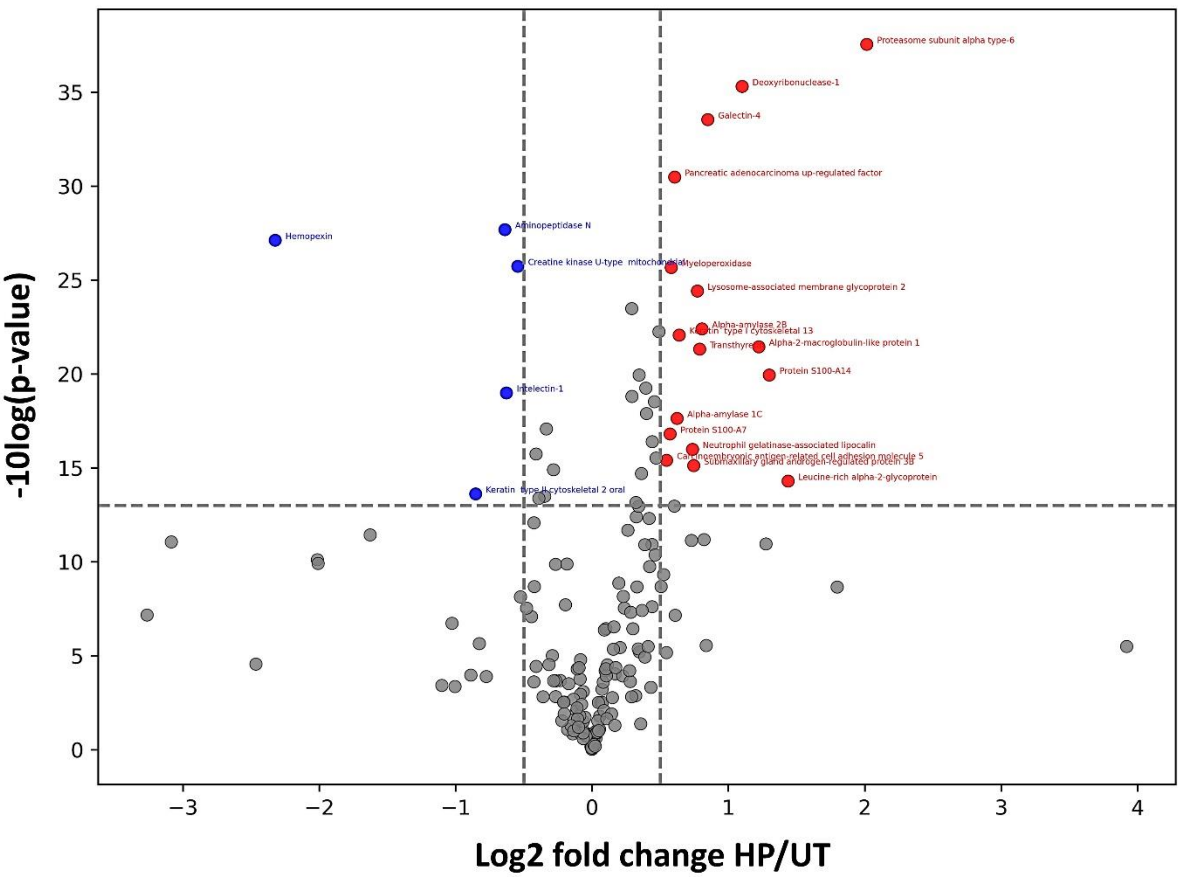

SI Figure 7. Proteins with significantly different abundance between Harnaschpolder (HP) and Utrecht (UT) wastewater samples (log_2 fold change > |0.5| and $p < 0.05$). Higher-abundance proteins included: carcinoembryonic antigen-related cell adhesion molecule 5, S100-A7, myeloperoxidase, pancreatic adenocarcinoma up-regulated factor, α-amylase 1C, keratin type I cytoskeletal 13, neutrophil gelatinase-associated lipocalin, submaxillary gland androgen-regulated protein 3B, lysosome-associated membrane glycoprotein 2, transthyretin, α-amylase 2B, galectin-4, deoxyribonuclease-1, α-2-macroglobulin-like protein 1, S100-A14, leucine-rich α-2-glycoprotein, and proteasome subunit α type-6. Lower-abundance proteins included: hemopexin, keratin type II cytoskeletal 2 oral, aminopeptidase N, intelectin-1, and creatine kinase U-type (mitochondrial). While abundance trends across sampling time points were not consistent, a few proteins showed a clear increase from Dec to March 2022 in the Utrecht samples (SI EXCEL DOC (worksheet "HS relative abundances"), including lactotransferrin, chymotrypsin-like elastase family member 3A, protein S100-A9, and zymogen granule membrane protein 16. However, a more accurate assessment of temporal trends would require a targeted approach with internal standards to eliminate biases introduced during sampling and sample preparation.
